# Therapeutic Applications of Nucleic Acids and Their Analogues in Toll-like Receptor Signaling

**DOI:** 10.3390/molecules171113503

**Published:** 2012-11-14

**Authors:** Vijayakumar Gosu, Shaherin Basith, O-Pil Kwon, Sangdun Choi

**Affiliations:** Department of Molecular Science and Technology, Ajou University, Suwon 443-749, Korea

**Keywords:** Toll-like receptor, nucleic acid analogues, cancer, autoimmune disease, viral infection, clinical status

## Abstract

Toll-like receptors (TLRs) belong to a family of innate immune receptors that detect and clear invading microbial pathogens. Specifically intracellular TLRs such as TLR3, TLR7, TLR8 and TLR9 recognize nucleic acids such as double-stranded RNA, single-stranded RNA and CpG DNA respectively derived from microbial components. Upon infection, nucleic acid sensing TLRs signal within endosomal compartment triggering the induction of essential proinflammatory cytokines and type I interferons to initiate innate immune responses thereby leading to a critical role in the development of adaptive immune responses. Thus, stimulation of TLRs by nucleic acids is a promising area of research for the development of novel therapeutic strategies against pathogenic infection, allergies, malignant neoplasms and autoimmunity. This review summarizes the therapeutic applications of nucleic acids or nucleic acid analogues through the modulation of TLR signaling pathways.

## 1. Introduction

The classification of immune responses as “innate” or “adaptive” is a basic concept of immunology. The innate immune system is the first line of defense against invading microbial pathogens, and the adaptive immune system is the second line of defense and affords protection against re-exposure to the same pathogen. However, innate sensing of pathogens leads to initiation of adaptive immunity [1]. Most metazoans rely on innate immunity, whereas innate and adaptive immunity overlap in vertebrates. One crucial difference between the innate and adaptive immune response is the innate immune response that is encoded in the germline DNA, while adaptive immune responses require gene rearrangements [1]. Innate immune recognition relies on a diverse set of germ line-encoded receptors, such as pattern recognition receptors (PRRs) expressed in the foot soldiers of the innate immune system, dendritic cells (DCs) and macrophages. Innate immune responses are triggered by PRRs that recognize molecules derived from microorganisms, such as pathogen-associated molecular patterns (PAMPs) and danger-associated molecular patterns (DAMPs, also known as damage-associated molecular patterns), thereby switching on the production of host defense genes including type I interferons (IFNs) [2,3,4,5]. PRRs are classified according to their structural homology: Toll-like receptors (TLRs), RIG-I-like receptors (RLRs), NOD-like receptors (NLRs), and C-type lectin receptors (CLRs). The TLR family is one of the largest and most well studied in terms of known ligands, downstream signaling pathways, and functional relevance [6,7,8,9,10].

TLRs are type I transmembrane receptors with an ectodomain comprised of leucine-rich repeats that mediate recognition of PAMPs, transmembrane domains, and intracellular Toll-interleukin (IL)-1 receptor (TIR) domains required for downstream signal transduction [8,11]. Ten human TLRs and 12 mouse TLRs have been identified, with TLR1-TLR9 being conserved in both species. Due to a retrovirus insertion, mouse TLR10 is nonfunctional and TLR11–13 are present in mice but lost from the human genome [12]. TLR1-2, TLR4-6, and TLR11-12 in mice and TLR10 in humans are expressed largely on the cell surface where they recognize microbial molecules such as flagellin protein [13] or lipid components [14,15,16]. The crucial role of TLRs in host defense is to regulate the innate and adaptive immune responses of epithelial cells at mucosal sites and mediate leukocyte recruitment to infected tissues [17,18]. Although the TLRs appear to have evolved as a warning system to detect infections, in some cases they can be triggered unexpectedly by self-molecules. This is well established for the intracellular TLRs that detect nucleic acids, including TLR3 (activated by double-stranded (ds) RNA), TLR7 and 8 (activated by single-stranded (ss) RNA), TLR9 (activated by CpG motifs within ssDNA) and recently discovered TLR13 (an orphan receptor in mice that detects conserved 23s ribosomal RNA) [19]. Thus, these TLRs act from the endosomal compartment to discriminate host and foreign nucleic acids; usually (not always), host DNA is excluded from the endosomal compartment. Vertebrate nucleic acids also have several modifications that functionally reduce the probability of activating intracellular TLRs. However, self RNA and DNA retain some ability to auto-activate TLR-driven immune responses. For example, several studies have revealed inappropriate activation of TLR7, TLR8, and/or TLR9 pathways by chromatin- or RNA-containing immune complexes leading to autoantibody production [20,21,22,23]. To avoid autoimmune reaction, nucleotide sensing TLRs must discriminate between microbial products and self-products; robust TLR7/9 responses to self-nucleotides predispose individuals to autoimmune diseases such as rheumatoid arthritis (RA) and systemic lupus erythematosus (SLE) [21,23]. Therefore, intracellular TLR responses must be tightly controlled to induce sufficient response against pathogens without inducing detrimental responses to self-products. This review focuses on nucleic acid involvement and summarizes therapeutic applications of nucleic acids or nucleic acid analogues in the TLR signaling pathway.

## 2. Nucleic Acid Sensing Toll-Like Receptors and Their Signaling Pathways

TLRs are a family of single membrane-spanning receptors expressed on sentinel cells of the immune system, including macrophages and DCs [24]. Activation of TLRs by specific ligand binding at the ectodomain induces receptor dimerization, the initial step in signal transduction. Other nucleic acid sensing receptors, including TLR3, TLR7/8, and TLR9 may be present as preformed inactive dimers and ligand binding may cause reorientation in the TIR domains [7,25]. In both cases, the TLR-TIR interaction serves as a platform for recruiting downstream signaling adapter proteins. The adapter protein acts via two signaling pathways: the myeloid differentiation primary response protein (MyD88)-dependent pathway and the TIR domain containing the adapter that induces the MyD88-independent pathway (also known as TRIF (TIR-domain-containing adapter-inducing interferon-β)-dependent pathway).

**Figure 1:**
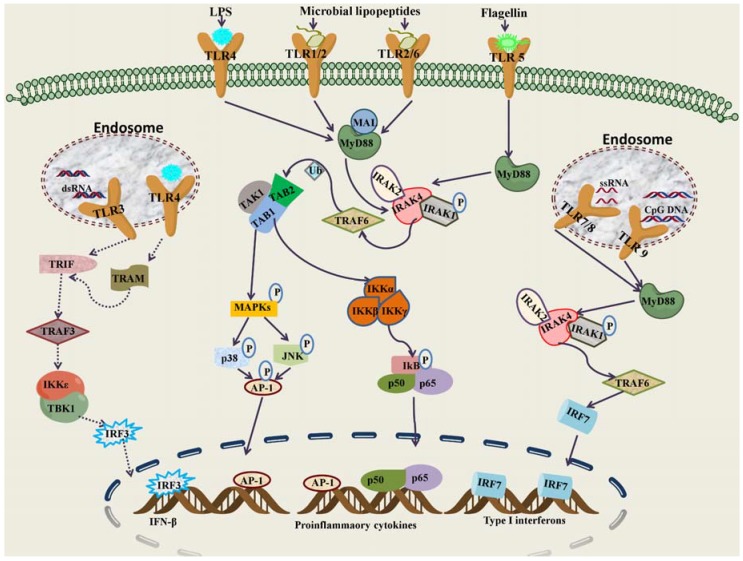
Toll-like receptor signaling networks. TLR1,TLR2, TLR4, TLR5, and TLR6 work at the extracellular surface and TLR3, TLR7, TLR8, and TLR9 function in the endosomal/lysosomal compartments. The activation begins with the cytosolic TIR domains of receptor and adaptor proteins. Upon stimulation in MyD88-dependent pathways, MyD88 recruits IRAK4 through the death domain interactions. IRAK4 phosphorylates IRAK1, which associates with TRAF6. TRAF6 activates the TAK1protein associated with TAB1 and TAB2. TAK1 phosphorylates the IKK complex, thereby activating NF-κB subunits, which translocate to the nucleus. TAK1 also activatesMAPKs, resulting in the phosphorylation of JNKand p38, which finally culminate in the activation of AP-1. AP-1 and NF-κB induce proinflammatory cytokines such as IL-1, IL-12, and TNF-α. In MyD88-independent pathways, TRIF induces IFN-β and IFN-inducible genes through the activation of TRAF3. Stimulation of TLR7, TLR8, and TLR9 induces IRF7, leading to the production of typeI IFNs. MAL: MyD88-adaptor like, Ub: Ubiquitin.

All TLRs, including TLR7, TLR8, and TLR9 depend on the MyD88-dependent pathway with the exception of TLR3, which exclusively uses the TRIF-dependent pathway to induce expression of proinflammatory cytokines [26]. Most TLRs activate the canonical signaling pathway through MyD88 [27], which in turn recruits and interacts with IL-1R-associated kinase-4 (IRAK4) [28]. IRAK4 phosphorylates IRAK1 and IRAK2 [29]; they in turn activate TNF receptor-associated factor 6 (TRAF6), which activates MAPKKK member TGF-β activated kinase 1 (TAK1) associated with TAK1-binding proteins 1 (TAB1) and TAB2. TAK1 phosphorylates and activates the inhibitor of kappa light polypeptide gene enhancer in B-cells kinase (IKK) complex, which in turn phosphorylates IκBs, resulting in their degradation and supporting the release and translocation of NF-κBs to the nucleus. Ultimately, NF-κB activation induces transcription of proinflammatory cytokines such as TNF-α, IL-6, IL-1, and IL-12, key mediators of the inflammatory response [30,31,32]. Unlike other TLRs, TLR3 can signal independently of MyD88 by associating with the adaptor protein TRIF [33]. TRIF associates with TRAF3, TRAF6, receptor interacting protein 1 (RIP1), and RIP3. The association of TRAF6 and RIP1 leads to activation of NF-κBs and MAPKs to induce proinflammatory cytokines. TRAF3 activation initiates the phosphorylation and activation of IRF3, leading to IFN-β production [34,35]. TLR4 signals through the same TRIF pathway; however, under most circumstances, it also requires the adaptor TRAM protein. TLR stimulation leads to the activation of other signaling pathways such as p38, JNKs, ERKs, IRFs (IRF3, IRF5, and IRF7), and MAPKs [36]. These pathways are essential for the orchestration of innate and adaptive immune responses and tissue repair in the host (Figure 1).

## 3. Cellular Localization and Regulation of Nucleic Acid Sensing TLRs

TLRs are type I transmembrane proteins that share a similar structural domain organization, including an extracellular domain for ligand recognition, transmembrane domain, and cytosolic domain. Thus, it is assumed that all TLRs are expressed on the cell surface. Several chimeric approaches demonstrated surface expression of TLR1, TLR2, TLR4, TLR5, and TLR6, whereas the nucleic acid sensing TLR3, TLR7, TLR8, and TLR9 localize within various intracellular compartments [37,38]. Though these TLRs are intracellularly localized, their function is initiated only within acidified endolysosomes; bafilomycin A1, chloroquine, and ammonium chloride prevent acidification and block TLR responses. Thus, internalized delivery of nucleic acids to the endolysosomes is critical for interaction with these TLRs [39]. TLR3, TLR7, and TLR9 share a common intracellular compartment [40]. Upon ligand stimulation, TLR7 and TLR9 rapidly move from the endoplasmic reticulum (ER) to the endolysosomes [41]. This translocation is associated with and regulated by 12-membrane-spanning ER protein UNC93B1 [41]. Under normal conditions, association of TLR9 with the N-terminal domain of UNC93B1 strongly activates TLR9 and suppresses TLR7 translocation, whereas TLR3 translocation is independent of N-terminal domain in UNC93B1 [41,42,43]. In a forward genetic screen of *N*-ethyl-*N*-nitrosourea mutants, a mouse ‘tripled D (3D)’ mutant of UNC93B1 was identified. TLR3, TLR7, and TLR9 signaling was abrogated while other TLRs were unaffected, and the mice were highly susceptible to microbial infection [44]. Absence of UNC93B1 is responsible for HSV-1 encephalitis in humans [45]. Therefore, UNC93B1 is necessary for strong intracellular TLR responses and for efficient translocation of each nucleotide sensing TLR to the endolysosomes. Gp96 and PRAT4 also reside in the ER and regulate TLR trafficking. Gp96 is a member of the heat shock protein 90 family and functions as a general chaperone for TLR1, TLR2, TLR4, TLR5, TLR7, and TLR9 [46]. Gp96-deficient macrophages exhibit defective cytokine induction in response to most TLRs [42]. PRAT4 regulates trafficking of TLR1, TLR2, TLR4, TLR7, and TLR9; however, it does not seem necessary for TLR3, therefore suggesting that trafficking of intracellular TLRs particularly TLR3 and TLR9 are regulated differently [47]. Full-length TLR9 proteins reside in the ER. Upon activation, the TLR9 ectodomain is cleaved by proteolytic enzymes, including various cathepsins and asparaginyl endopeptidase [48,49,50,51,52]. Therefore, biologically active TLR is restricted to endolysosomes and initiates signal transduction by sensing CpG DNA. There is uncertainty regarding the functional cleavage of TLR9, as deletion of the N-terminal leucine-rich repeats makes TLR9 ligand unresponsive [53]. TLR7 is also cleaved and TLR3 is cleaved by cathepsins B and H [54].

## 4. Nucleic Acid Sensing TLR Agonists and Their Therapeutic Applications

Nucleic acids from microbial pathogens are agonists for intracellular TLRs; however, these nucleic acids display certain features that permit discrimination from host nucleic acids, such as dsRNA, 5'-triphosphate RNA, and unmethylated CpG DNA, that rectify innate immune responses and are beneficial in the treatment of inflammatory diseases, infection, cancer, and allergies. Intracellular TLRs, particularly TLR7 and TLR9, are involved in various autoimmune diseases by sensing immune complexes such as small ribonucleotide proteins comprised of self-nucleotides; an association between inflammation and cancer development has long been appreciated [55]. Given the central role of molecules in the intracellular TLR pathways in the innate immune response, researchers have focused on identifying synthetic agonists and antagonists to enhance the immune response. Generally, synthetic agonists or antagonists are developed based on natural TLR agonists; development of synthetic stimulatory motifs may be a promising approach in modulating the immune response via intracellular TLRs.

### 4.1. TLR3

TLR3 is specific to viral RNA and can be found in a wide range of cells, including epithelial cells, dendritic cells, monocytes, mast cells, and natural killer (NK) cells. TLR3 recognizes polyinosinic: polycytidylic acid (poly I:C), a synthetic analogue of dsRNA that mimics viral infection and induces antiviral immune responses by triggering production of type I IFN and inflammatory cytokines. In addition to poly I:C, TLR3 also recognizes ssRNA polyinosinic acid (Poly I) resulting in the activation of B lymphocytes, DCs and macrophages [56]. TLR3 recognizes dsRNA from dsRNA viruses or dsRNA produced during replication of ssRNA or DNA viruses. TLR3 null mice are susceptible to ssRNA viruses, including Semliki Forest virus, West Nile virus (WNV), and encephalomyocarditis virus (EMCV) as well as DNA viruses such as mouse cytomegalovirus (MCMV) and HSV-1 [57,58,59,60]. TLR3 activation may be a promising approach for anticancer therapy and vaccine adjuvancy. TLR3-dependent IFN-β production induced by poly(I:C) inhibits growth of clear cell renal cell carcinoma and breast cancer cells [61]. TLR ligands as adjuvants provide advantages in selectively expressing TLR3 and subsequent immune responses such as type I IFN, cytokine/chemokine production, DC maturation, and cytotoxic T-lymphocytes and NK cell activation. Thus, poly(I:C) is being considered as an adjuvant for a number of vaccines, including Neisseria meningitidis serogroup B, human immunodeficiency virus (HIV) gag, *Plasmodium falciparum*, circumsporozoite, *Mycobacterium tuberculosis*, and tumor-associated proteins [62,63,64,65]. Addition of mismatched uracil and guanosine to poly(I:C) produces polyI:polyC_12_U (trade name: Ampligen^®^; generic name: rintatolimod) that induces Th1 (type 1 helper T cell) cytokine IL-12 with less IL-10 production and is effective in healthy subjects and cancer patients. *In vivo* Ampligen^®^ was shown to inhibit the growth of a large panel of neoplasms, in both immunodeficient [66,67] and immunocompetent models [68,69]. Ampligen^®^ is an antiviral biological response modifier developed for treatment of HIV, influenza, chronic fatigue syndrome, and hepatitis B and C infection [70,71]. The safety, toxicity, and intravenous infusion clinical trials (phase I) were recently completed in HIV patients (NCT00000735 and NCT00000713). Phase III clinical trials are ongoing on chronic fatigue syndrome (NCT00215813). Ampligen^®^ targets EGFR and very effectively destroys EGFR-overexpressing tumors with no adverse or toxic effects [72], suggesting that tumor therapeutics might be possible with TLR ligands. Ampligen^®^ is in clinical trials in combination with autologous tumor cell lysate (Phase I-II) for peritoneal cancer (NCT01312389); in a vaccine therapy for HER2 breast cancer (NCT01355393); and in combination with IFN and celecoxib in resectable colorectal cancer (NCT01545141).

Another synthetic agonist of TLR3 is poly(A:U), which activates dendritic cells and T lymphocytes. Poly(A:U) promotes antigen-specific Th1 immune responses and boosts antibody production [73]. Immune adjuvant effects through TLR3 and TLR7 can be achieved with systemic administration of poly(A:U); TLR3 is required to generate IFN-γ–producing CD8+ T cells, and TLR3 and TLR7 are required for clonal expansion of antigen-specific cells [74].The potent adjuvant activity of poly(A:U) has been exploited in breast cancer cells [75]. During the past three decades, poly(A:U) has proven to be efficient for adjuvant therapy of various cancers, including gastric cancer, resectable colorectal carcinoma, and breast cancer [76,77,78]. Poly(A:U) is not currently undergoing clinical trials. Hiltonol^®^ is a synthetic polyriboinosinic-polyribocytidylic acid (poly I:C) condensed with poly-L-lysine and carboxymethyl cellulose (LC), a potent immunomodulating agent. It exhibits antiviral activity via induction of α-, β-, and γ-IFN *in vivo* [79]. However, no antitumor efficacy was observed in patients with metastatic melanoma [80]. The safety and efficacy of this compound are being investigated in about 20 phase I/II clinical trials. Several clinical trials of poly-ICLC with DC vaccine peptides are ongoing for various advanced malignancies such as glioma and prostate cancer (NCT01188096, NCT00773097, NCT01079741, and NCT00374049). IPH-3102 is another specific TLR3 agonist with high molecular mass that mimics dsRNA, activates NF-κB and induces type I IFN responses *in vitro*, thereby exerting cytotoxic effects against melanoma and carcinoma cells. Moreover, IPH-3102 consistently mediates immunostimulatory effects *in vivo* in mice [81,82]. The recent clinical status of TLR3 agonists is shown in Table 1.

**Table 1:** Clinical status of TLR3-recognizing nucleic acid analogues.

Compd.	Phase	Status	Indications	Notes	ClinicalTrials.gov [83]
Ampligen^®^	I	Completed	HIV infections	Single agent	NCT00000735
	I	Completed	HIV infections	Single agent	NCT00000713
	III	Recruiting	Chronic fatigue syndrome	Single agent	NCT00215813
		Not yet recruiting	Colorectal cancer	Combined with IFN-α and celecoxib	NCT01545141
	I-II	Active but not yet recruiting	Ovarian, fallopian tube or primary peritoneal cancer	Combined with tumor cell lysate and multiple adjuvants	NCT01312389
	I-II	Recruiting	Breast cancer	GM-CSF as a combined adjuvant strategy with HER2 vaccination	NCT01355393
Hiltonol^®^	I	Completed	Respiratory infections	Single agent	NCT00646152
	II	Recruiting	Low grade glioma	Single agent	NCT01188096
	II	Recruiting	Colorectal cancer	Combined with MUC1peptide vaccine	NCT00773097
	I-II	Recruiting	Melanoma	Combined with NY-ESO-1based vaccine	NCT01079741
	I	Active but not yet recruiting	Prostate cancer	Combined with MUC1	NCT00374049
	0	Recruiting	Breast cancer	Combined with MUC1 peptide vaccine	NCT00986609
	0	Recruiting	Adenocarcinoma	Vaccination with DCs	NCT01677962
	I	Not yet recruiting	Melanoma	Combined with multipeptide vaccine	NCT01585350
	II	Recruiting	Advanced myeloma	Combined with multipeptide vaccine and lenalidomide	NCT01245673

### 4.2. TLR7/TLR8

TLR7 and TLR8 recognize ssRNA from viruses such as influenza, VSV, and Newcastle disease virus [84,85]. TLR7 and TLR8 share sequence similarity and recognize the same native pathogen. Furthermore, a recent report has shown that ssORN can activate TLR7, TLR8 and TLR9 pathways [86]. Recognition sequence specificity for TLR7 and TLR8 has not been defined as it has for TLR9 (hexameric CpG); however, ssRNA sequences containing GU-rich or poly-U sequences can trigger both receptors. TLR7 preferentially recognizes GU-rich RNA sequences and TLR8 has more affinity for AU-rich sequences [84,87,88,89]. Human TLR7 and TLR8 respond to synthetic ssRNA and certain synthetic nucleoside analogues, such as guanosine-containing compounds and imidazoquinoline. Additionally, TLR8 stimulation inhibits TLR7 response [90]. However, some of the TLR7 agonists used in clinical trials has shown to trigger TLR8 response [91]. The intracellular localization of TLR7 and TLR8 usually necessitates cellular uptake of these receptors.

Imiquimod [R837: 1-(2-methylpropyl)-1*H*-imidazo[4,5-c]quinolin-4-amine], the first member of the new class of immune response modifiers, is a nucleoside analogue that stimulates the innate immune response via the induction, synthesis and release of specific cytokines. The induction of IFN-α, IL-6 and tumor necrosis factor (TNF)-α by imiquimod has been observed *in vitro* and in both human and animal studies [91,92,93]. Imiquimod also affects other aspects of the innate response in animal models, such as NK cell activity, activation of macrophages to secrete cytokines and nitric oxide and induction of B lymphocytes to proliferate and differentiate [94]. This drug was approved in 1997 for the topical treatment of external genital warts caused by human papillomavirus (HPV); however it is also effective for other HPV-associated warts such as nongenital warts, molluscum contagiosum, genital herpes, and squamous cell carcinoma (SCC) [95]. Imiquimod is the first approved TLR7 agonist. It has been widely used in both infectious and neoplastic cutaneous diseases. It is effective against primary skin tumors and skin metastasis when used for the treatment of cancer [96]. Imiquimod provides improvements in basal cell carcinoma, actinic keratosis, malignant melanoma, cutaneous T-cell lymphoma, and cutaneous extra-mammary Paget’s disease [96]. Topical imiquimod is undergoing phase II clinical trials with Abraxane^®^ to investigate side effects in breast cancer patients (NCT00821964). Resiquimod [R848; 4-amino-2(ethoxymethyl)-α,α-dimethyl-1*H*-imidazol[4,5-c]quinoline-1-ethanol], another structurally similar compound, stimulates immune responses via TLR7 in mice and via TLR7 and TLR8 in humans [97,98]. Resiquimod triggers significant cytokine secretion, macrophage activation, and enhancement of cellular immunity [91]. Resiquimod was developed for antiviral treatment of genital herpes; however, development was suspended due to lack of significant therapeutic efficacy [99]. Resiquimod is now being evaluated in clinical trials as a single agent or in combination with vaccination strategies for actinic keratosis (NCT01583816), melanoma (NCT00960752), and cutaneous T-cell lymphoma (NCT01497795). CL097 is a derivative of resiquimod that activates both TLR7 and TLR8. It induces NF-κB activation at 0.4 µM in TLR7-transfected cells and at 4 µM in TLR8-transfected cells [100,101]. The selective TLR7 agonist 852A (3M-001) is structurally related to imiquimod and stimulates plasmacytoid dendritic cells (pDCs) to produce cytokines such as IFN-α, IL-1 receptor antagonist, and IFN-inducible protein-10 [102]. Clinical trials of this compound as a single agent have been completed for cutaneous melanoma (NCT00189332), refractory solid tumors (NCT00095160), and high-grade dysplasia in Barrett’s Esophagus (NCT00386594). CL075 (3M-002) is another compound derived from thiazoquinoline that triggers TLR8 in human peripheral blood mononuclear cells (PBMCs). Agonistic activity of CL075 at TLR8 activates NF-κB and triggers secretion of TNF-α and IL-12 [103]. This compound may also activate TLR7, thereby inducing section of IFN-α, however, to a lesser extent. Another immunostimulatory variant of imiquimod is 3M-003, which activates both TLR7 and TLR8. Gardiquimod [1-(4-amino-2-ethylaminomethylimidozo[4,5-c]quinolon-1-yl)-2-methylpropan-2-ol] is a selective agonist of human and mouse TLR7, similar to imiquimod. This compound induces activation of NF-κB in cells expressing human or mice TLR7 [104] and is more potent than imiquimod. At higher concentration, it is also known to activate TLR8.

Stimulation of TLR7/TLR8 can be achieved with base analogues such as purine and pyrimidine derivatives. Loxoribine is a guanosine ribonucleoside analogue that activates human and mouse TLR7. This compound exhibits anti-tumor and anti-viral activity via activation of NK and B cells to induce multiple cytokines [105,106,107]. Bropirimine is an aryl pyrimidinone analogue, an antineoplastic compound that induces IFN-α and is used for treatment of carcinoma [108,109]. The mechanism of action of bropirimine is likely to be direct anti-tumor effect rather than cytokine mediated anti-tumor activity [110]. Expression of TLR7 occurs in normal and hepatitis C virus (HCV)-infected hepatocytes, and TLR7 activation itself reduces levels of HCV-mRNA [111]. Isatoribine (ANA 245) and ANA 975 (an oral prodrug of isatoribine) are guanosine nucleoside analogues and specific TLR7 agonists developed for treatment of HCV infection [112]. However, ANA 975 has been discontinued due to unacceptable toxicity [113]. ANA773 (NCT01211626) is another oral TLR7 agonist prodrug that induces IFN-α. This compound may have utility in chronic HCV infection [114]. AZD8848/DSP-3025 is a novel TLR7 agonist being tested for the treatment of asthma and hay fever. Initial clinical trials to investigate safety and efficacy in allergic asthma patients challenged with inhaled allergen have been completed; however, results have not been published (NCT00688779, NCT00999466, and NCT01185080). To investigate the pharmacokinetic and pharmacodynamic properties of AZD8848/DSP-3025, phase I clinical trials in healthy volunteers with a single ascending dose are underway (NCT01560234).

**Figure 2:**
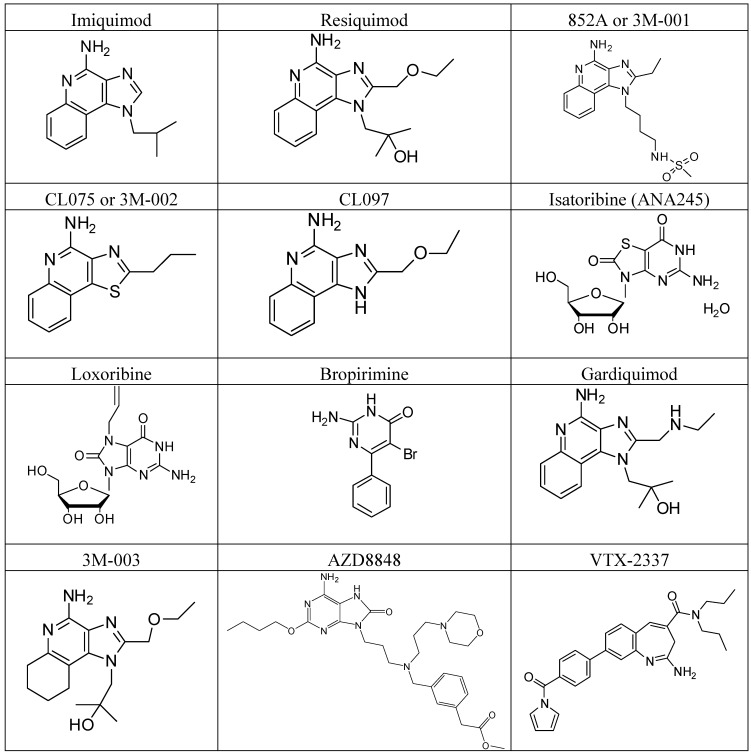
TLR7 and TLR8-recognizing synthetic nucleoside analogues.

VTX-2337 and VTX-1463 are selective small molecule TLR8 agonists; VTX-2337 (NCT00688415) stimulates myeloid dendritic cells and monocytes and enhances NK cell responses, whereas VTX-1463 reduces allergic responses by suppressing the Th2 (type 2 helper T cell)-mediated response. VTX-2337 is being investigated in advanced recurrent head and neck squamous cell carcinoma (HNSCC) patients in combination with cetuximab (NCT01334177), and with doxorubicin for the treatment of peritoneal cavity cancer (NCT01294293). VTX 378, a novel TLR8 agonist that interacts with monocytes, macrophages, and myeloid dendritic cells, may be administered via nasal spray directly to allergic sites, where it may initiate an anti-allergic response. The molecular structures of TLR7/8 synthetic nucleoside analogues are shown in Figure 2. The recent clinical status of TLR7/8 agonists is shown in Table 2.

**Table 2:** Clinical status of TLR7/8-recognizing small molecule nucleoside analogues.

Compd.	Phase		Status		Indications	Notes		ClinicalTrials.gov [83]
Imiquimod	IV		Recruiting		Basal cell carcinoma	Single agent		NCT00803907
	Not available		Recruiting		Melanoma	Combined with multipeptide vaccine		NCT01264731
	II		Recruiting		Breast cancer	Together with Abraxane		NCT00821964
		I	Recruiting	Glioma		Combined with lysate based vaccine	NCT01400672	
		I-II	Recruiting	Metastatic breast cancer		Together with radiotherapy	NCT01421017	
		IV	Recruiting	Plantar warts			NCT01059110	
		Not available	Recruiting	Psoriasis		Together with UV radiation	NCT00470392	
Resiquimod		II	Recruiting	Actinic keratosis		Single agent	NCT01583816	
		I-II	Recruiting	T cell lymphoma		Single agent	NCT01676831	
		I-II	Recruiting	T cell lymphoma		Single agent	NCT01497795	
		II	Recruiting	Melanoma		Combined with gp 100 and MAGE-3 based vaccine	NCT00960752	
		I-II	Active but not recruiting	Advanced malignancies		Combined with CDX-1401	NCT00948961	
VTX-2337		I	Recruiting	HNSCC		Combined with cetuximab	NCT01334177	
		I	Recruiting	Reproductive tract cancer		Combined with liposomal doxorubicin	NCT01294293	
		II	Not yet recruiting	Reproductive tract cancer		Combined with liposomal doxorubicin	NCT01666444	
		I	Completed	Advanced solid tumor		Single agent	NCT00688415	
852A		II	Completed	Melanoma		Single agent	NCT00189332	
		I	Completed	Neoplasms		Single agent	NCT00095160	
		Not available	Terminated	Barrett esophagus		Single agent	NCT00386594	
		II	Terminated	Hematologic malignancies		Single agent	NCT00276159	
ANA773		I	Completed	HCV infection		Single agent	NCT01211626	
AZD8848/DS P-3025		II	Completed	Allergic asthma		Single agent	NCT00999466	
		I	Completed	Allergic rhinitis		Single agent	NCT00688779	
		I	Recruiting	Healthy		Single agent	NCT01560234	
		I	Completed	Allergic rhinitis		Single agent	NCT00925678	

### 4.3. TLR9

TLR9 localizes within the endosomal compartment of monocytes, macrophages, B cells, and pDCs. In humans and mice, TLR9 is largely expressed in pDCs, which are crucial for the production of IFNs in response to viral infections and for regulation or eradication of infected cells [115]. TLR9 detects unmethylated deoxycytidylate-phosphate-deoxyguanylate (CpG) motifs, which are relatively common in viral and bacterial DNA, but are uncommon in vertebrate DNA; if present, they are highly methylated [116]. CpG oligodeoxynucleotide (ODN) is a synthetic TLR9 agonist resembling microbial DNA, as they have a complete or partial phosphorothioate backbone instead of a typical native phosphodiester bond. TLR9 activation by CpG ODN induces potent Th1-type innate and adaptive immune responses with prominent release of IFN-α, IL-12, and IL-18. Targeting TLR9 with CpG ODN has been extensively studied for the treatment of cancer and various infectious diseases [117]. The immune responses of CpG depend on the length of the nucleotide sequence, nature of the backbone, and presence of specific structural motifs. CpGs are classified into class A, B, or C based on the cytokine profiles they induce [117]. Class A members have a naturally occurring phosphodiester backbone with poly CpG motifs at the center. Class B CpG ODN contains a phosphorothioate backbone throughout the sequence and induces B cell activation and proliferation. Class C has 1 or 2 CpG motif(s) with a phosphodiester backbone at the 5' end, and contains a palindromic sequence on a phosphorothioate backbone at the 3' end. It is worth noting that ectopically expressed TLR9 in the plasma membrane detects microbial and host DNA [118], demonstrating that intracellular localization, not ligand properties, is the basis for discrimination between self- and non-self DNA. As an adjuvant for vaccines and as mono- or combination therapies, CpG ODNs have considerable potential for the treatment of cancer and infectious and allergic diseases [119]. They also have demonstrated substantial effects in rodent and primate models of asthma and allergic diseases with encouraging results in some early human clinical trials [120].

The immune modulatory oligonucleotides (IMOs) which contain CpR, YpG, and R’pG stimulatory motifs are being developed as second generation agonists for TLR9 signaling. IMO-2055 (EMD1201081), a synthetic CpR oligonucleotide mimic of unmethylated CpG sequences in bacterial DNA, is a potent TLR9 agonist [121]. This compound induces innate and adaptive immune responses and exhibits potent antitumor activity in murine models of colon carcinoma and melanoma when used as a monotherapy [122]. However, when used in combination with chemotherapeutic agents, its activity is improved. This has been investigated in clinical trials with Erbitux^®^ (an inhibitor of epidermal growth factor, EGFR) and FOLFIRI in colorectal cancer (NCT00719199) or with Erbitux^®^, 5-fluorouracil (5-FU), and cisplatin in patients with recurrent/metastatic squamous cell carcinoma of the head and neck (SCCHN) (NCT01360827), and with erlotinib and bevacizumab in non-small-cell lung carcinoma (NSCLC) (NCT00633529). Safety and efficacy of IMO-2055 are being investigated as a single agent in metastatic or locally recurrent clear cell renal carcinoma (NCT00729053) and for the treatment of second-line cetuximab-naïve subjects with recurrent or metastatic SCCHN in combination with Erbitux^®^ (NCT01040832). IMO-2125 is a novel TLR9 agonist developed for the treatment of chronic HCV infection. This compound activates T cells and NK cells and induces sustained levels of IFN-α and other cytokines with potent anti-HCV activity in non-human primates [121]. IMO-2125 phase I clinical trials, one as a single agent in null responders with HCV (NCT00728936), the other in combination with ribavirin and IMO-2125 in naive hepatitis C infection (NCT00990938), are completed; however, official results have not yet been disclosed. A TLR9 agonist, IMO-2134 (also known as QAX-935), is a lead compound for asthma and allergy indications. Intranasal administration in monkeys induces several IFN-dependent genes and does not increase airway resistance or inflammation [123]. Idera is evaluating the development of IMO-2134.

MGN-1703 and MGN-1706 are novel TLR9 agonists that contain a phosphodiester backbone and fold in a dumbbell-like structure known as double stem-loop immunomodulators (dSLIMs). Each of these compounds has anticancer effects in preclinical models; they target TLR9 receptors in certain immune cells. *In vivo* dSLIMs enhance therapeutic efficacy in leukemia when combined with granulocyte monocyte colony-stimulating factor (GM-CSF) [124]. The dSLIM-activated immune system can overcome its fatal tolerance of cancer cells. The MGN-1703 clinical trial is in the phase II evaluation of efficacy and safety of maintenance therapy versus placebo control in patients with advanced colorectal carcinoma (NCT01208194). Immunostimulatory DNA sequences (ISS) composed of unmethylated short CpG dimers can induce IFN and IFN-inducible proteins via antigen-presenting cells [125]. ISS activation of TLR9 stimulates production of Th1 cells and Th1 response. ISS is linked with antigens or used alone to suppress the Th2 response. ISS-1018 is a 22-bp single-stranded phosphorothioate oligonucleotide that induces production of immunoglobulin and IFN-α by B cells and IFN-β, IL-12, and TNF-α by pDCs [126]. ISS-1018 is in clinical trials alone and combination with antigens to determine efficacy and safety in non-Hodgkin’s lymphoma, cancer, and allergy. This compound has also been extensively studied as an adjuvant hepatitis B virus vaccine HEPLISAV^TM^. So far clinical trials have shown that HEPLISAV™ produces rapid, high titer and sustained seroprotection in healthy adults and vaccine hyporesponsive populations [127]. Currently two phase III clinical evaluation of HEPLISAV^TM^ is ongoing for renal disease. HEPLISAV^TM^ demonstrated superior seroprotection in diabetic patients when compared to currently available vaccine Engerix-B [128] (NCT00426712, NCT00498212, NCT01282762, NCT00435812, NCT00511095, NCT00985426, NCT01195246, and NCT01005407). Furthermore, ISS-1018 was effective in combination with rituximab (a chimeric monoclonal antibody against CD20) in B cell follicular non-Hodgkin’s lymphoma in a phase II clinical trial (NCT00251394). ISS-1018 combined with Irinotecan and Erbitux is in phase I trials for metastatic colorectal carcinoma (NCT00403052). An evaluation of the safety and efficacy of TOLAMBA (TOLAMBA consists of 1018-ISS linked to the purified major allergen of ragweed called Amb a 1) is being investigated for ragweed-allergic rhinitis and is in phase II trials (NCT00537355). SD-101 is another novel C-type TLR9 agonist being developed for chronic HCV infection [129]. The safety, tolerability, and efficacy of this drug as a single agent or in combination with ribavirin has been tested in HCV in phase I trials (NCT00823862 and NCT00599001). *In vitro* data has shown SD-101 induces 20-fold higher levels of IFNs than first generation TLR9 agonists and provides potent anti-HCV activity [130].

CpG 7909 (PF-3512676) and CpG 685 (GNKG168) are immunomodulating synthetic oligonucleotides (class B) that are 18–28 nucleotides in length with a phosphorothioate backbone; they stimulate B cells and monocytes [131]. CpG 7909 specifically targets TLR9, stimulates human B cell proliferation, enhances antigen-specific antibody production, and induces IFN-α production, IL-10 secretion, and NK cell activity. In humans, subcutaneous administration of CpG 7909 activates myeloid and pDCs, producing an effective Th1 response that can enhance anticancer immunity. This compound has been evaluated alone and in combination with vaccine adjuvants in many phase I/II clinical trials in various tumor types, including renal cell carcinoma, lymphoma, glioblastoma, melanoma, esophageal squamous cell carcinoma, NSCLC, and leukemia [132,133]. CpG 7909 is being developed for use in metastatic breast cancer in combination with Herceptin^®^ (a monoclonal antibody that interferes with the HER2/neu receptor) (NCT00031278); for non-Hodgkin’s lymphoma in combination with rituximab and yttrium Y 90 ibritumomab tiuxetan drugs (NCT00438880), and for B cell lymphoma, in combination with radiotherapy. Immunization with MAGE-3.A1 peptide mixed with CpG 7909 adjuvant in metastatic melanoma (NCT00145145) and esophageal cancer (NCT00669292), and immunization with NY-ESO-1 protein mixed with CpG 7909 in prostate cancer patients are being clinically developed. Sepsis disease is being evaluated in phase I clinical trials with or without CpG 7909 adjuvant (NCT01164514). The phase I clinical trial has been completed for the malaria vaccine candidate CpG 7909 with Alhydrogel^®^ (NCT00427167). Along with the numerous studies of CpG 7909, the safety and efficacy of CpG 685 is being evaluated in phase I clinical trials for relapsed or refractory B-cell chronic lymphocytic leukemia (B-CLL) (NCT01035216). AV7909 is a new vaccine, which comprises Biothrax^®^ with CpG 7909. The phase I clinical trials investigating safety and efficacy of this compound are completed (NCT01263691) [134]. AVE0675 and SAR 21609 (CpG ODNs) are being developed for the treatment of asthma and viral respiratory tract infections either alone or in combination with specific allergen immunotherapy [135]. DIMS 0150 is lead drug completing its third phase II clinical trial for the treatment of steroid-resistant/dependent ulcerative colitis [136]. The recent clinical status of TLR9 agonists are shown in Table 3.

**Table 3:** Clinical status of TLR9-recognizing nucleic acid analogues.

Compd.	Phase	Status	Indications	Notes	ClinicalTrials.gov [83]
IMO-2055	I	Terminated	Colorectal cancer	Combined with Erbitux and FOLFIRI	NCT00719199
	I	Terminated	Head and neck carcinoma	Combined with Erbitux, 5-FU, and cisplatin	NCT01360827
	II	Active but not recruiting	Head and neck carcinoma	Combined with Erbitux	NCT01040832
	II	Completed	Renal cell carcinoma	Single agent	NCT00729053
	I	Completed	Non-small cell lung cancer	Single agent	NCT00633529
IMO-2125	I	Completed	Hepatitis c	Combined with ribavirin	NCT00990938
	I	Completed	Hepatitis c	Single agent	NCT00728936
MGN-1703	II	Active but not recruiting	Colorectal carcinoma	Single agent	NCT01208194
HEPLISAV^TM^	III	Active but not recruiting	Chronic kidney disease	Single agent	NCT00985426
	III	Recruiting	Renal disease	Single agent	NCT01195246
SD-101	I	Completed	Hepatitis c	Combined with ribavirin	NCT00823862
CpG 7909	II	Recruiting	Breast cancer	Combined with trastuzumab	NCT00824733
	I-II	Completed	Metastatic breast carcinoma	Combined with Herceptin	NCT00031278
	I-II	Terminated	Malignant melanoma	Combined with MAGE-3.A1 peptide	NCT00145145
	I	Suspended	Septicemia	Combined with J5-OMP vaccine	NCT01164514
GNKG168	I	Recruiting	Leukemia	Single agent	NCT01035216

## 5. Nucleic Acid Sensing TLR Antagonists and Their Therapeutic Applications

In addition to their potential as a target for adjuvants, nucleic acid sensing TLRs have been strongly implicated in autoantibody production. It is probable that intracellular TLRs can be activated by immune complexes containing self-DNA or self-RNA, thereby leading to the development of autoimmune diseases such as SLE and RA [21,23]. Therefore, inhibition of intracellular TLR activation by a synthetic compound is a promising research area for the treatment of autoimmune diseases. Intracellular antagonists are compounds developed as structural agonist analogues that bind to the receptor but fail to activate the TLRs, thus suppressing the immune response responsible for induction of the autoimmune/inflammatory cascade.

### TLR7, TLR8, and TLR9

Overexpression and deregulation of TLRs initiate many inflammatory and autoimmune diseases. Among the intracellular TLRs, TLR7 and TLR9 play a crucial role in the activation of autoreactive B cells, and subsequent development of autoimmune diseases such as SLE. The elevated level of IFN-α associated with SLE recognizes nucleic acid-containing immune complexes [137,138]. Current SLE treatments include antimalarial drugs such as hydroxychloroquine (HCQ), a TLR9 antagonist; however, it is a lesser antagonist of TLR7 and TLR8. This compound is undergoing phase III clinical trials for the treatment of dry eye in patients with primary Sjogren's syndrome (NCT01601028). Most of the derivatives and small molecule analogues of chloroquine and quinacrine reduce activation of the immune response [139].

CpG 52364 is a small molecule TLR antagonist specifically developed for TLR7, TLR8, and TLR9 to inhibit progression of SLE and other autoimmune diseases at an early stage by blocking inappropriate TLR activation [140]. The drug completed the phase I clinical trial for safety in humans (NCT00547014). IMO-3100 is a novel DNA-based antagonist of TLR7 and TLR9 for the treatment of SLE, RA, multiple sclerosis, psoriasis, and colitis. TLR7- and TLR9-mediated cytokines are critical factors in autoimmune disease. The compound is specifically designed to suppress the key cytokines by inhibiting TLR7 and TLR9 activity; however, some autoimmune disease treatments specifically target inhibition of individual cytokines. IMO-3100 is in a phase II clinical trial for patients with moderate-to-severe plaque psoriasis (NCT01622348). IMO-8400 is another novel DNA-based antagonist of TLR7, TLR8, and TLR9, which inhibits TLR7- and TLR9-mediated immune responses in mice, and TLR7-, TLR8-, and TLR9-mediated immune responses in human and non-human primates. IMO-8400 is in development for treatment of autoimmune diseases. Idera expects to submit an investigational new drug (IND) application for IMO-8400 to the Food and Drug Administration during the fourth quarter of 2012, and has selected lupus as the initial indication for clinical development [141].

Endosomal TLR inhibitors are a novel class of oligonucleotides called immunoregulatory sequences (IRS) that specifically inhibit the TLR-stimulated cytokines responsible for autoimmune and inflammatory disease. IRS blocks IFN-α and reduces symptoms in multiple autoimmune disease models such as lupus, inflammatory skin disorders, and RA. IRS 954 (DV-1709) is a specific inhibitor of TLR7 and 9 that reduced serum levels of nucleic acid-specific antibodies, and reduced end-organ damage in a lupus murine model [142]. Treatment of HIV-induced PBMCs with this compound reduced IFN-α production, suggesting therapeutic opportunity for HIV infection [143].

## 6. Nucleic Acid Sensing Toll-Like Receptors in Autoimmunity

Endosomal TLR3, TLR7, TLR8, and TLR9 differentiate between host- and pathogen-derived nucleic acid. However, inappropriate activation of specific regulators contributes to autoimmune diseases such as SLE and RA [144]. TLR7 and TLR9 respond to host-derived RNA and DNA, respectively. In general, extracellular DNase and RNase degrade host-derived nucleotides [145] before reaching the TLRs in the endosomes. Some data suggest SLE leads to loss of DNase I function [145]. In autoimmune conditions, nucleotide-binding proteins such as autoantibodies, antimicrobial peptides, and high-mobility group box 1 (HMGB1) transport self-derived nucleotides to endolysosomes. Small ribonucleotide proteins bind self-RNA molecules and trigger TLR7 to induce type I IFN, inducing autoimmunity [146]; in general, type I IFNs inhibit pathogen replication. Increased type I IFN is associated with disease severity in more than half of SLE patients [147,148]. Increased levels of nucleotide antibodies contribute to the loss of innate tolerance. Antimicrobial peptides LL-37 (human member of the cathelicidin family of antimicrobial peptides) and HMGB1 are important for self-DNA internalization in endosomes [149]. The self-DNA associated with LL-37 results in robust initiation and production of type I IFN. Overexpression of LL-37 mediates DC activation in psoriasis, an inflammatory disease of the skin [149,150]. HMGB1 is not secreted from apoptotic cells, but is released in abundance from necrotic cells due to induction of proinflammatory cytokine; HMGB1 associates with DNA such as CpG [151]. TLR9 activation by receptor for advanced glycation end products (RAGE) with HMGB1 complex stimulates robust production of type I IFNs, which activate antibody-producing plasmatocytes [152]. The Yaa genetic modifier experiment also indicated that overexpression of TLR7 contributes to the development of SLE [153]. Reports on TLR9 suggested a pathogenic role for TLR9 in psoriasis, lupus nephritis, adjuvant-induced arthritis, and a mouse model of multiple sclerosis [154]. TLR9-deficient mice developed several clinical diseases, including glomerulonephritis, a shortened lifespan, and in a few models, increased autoantibodies to RNA-associated autoantigens [155]. In addition, TLR9 ligand prevents inflammatory arthritis [156].

## 7. Conclusions

Nucleic acid sensing TLRs provide immunity to infection and adjuvanticity, and reduce chronic inflammation. Agonists of intracellular TLRs such as TLR3 agonist poly(I:C) and TLR7/8 agonist imidazoquinoline promote activation of Th1 immune responses; the TLR9 synthetic agonists are being investigated for cancer immunotherapy, asthma and allergy therapy, and vaccine adjuvants. Imiquimod has been approved for topical treatment of genital warts, actinic keratosis, and superficial basal cell carcinoma, although resiquimod was developed for antiviral treatment but was suspended due to lack of therapeutic efficacy. It is now being evaluated for actinic keratosis, melanoma, and cutaneous lymphoma. In addition, intracellular TLR antagonists have shown to be more effective in the treatment of autoimmune diseases. So far therapeutic targeting of TLRs in several diseases using TLR agonists has shown to be profoundly successful in both clinical and preclinical trials. However, recent reports have shown that the TLR agonists can promote cancer cell survival, migration and tumor progression. For example TLR7/8 agonists were shown to increase tumor viability and metastasis in lung cancer cells [157] and TLR3 agonists shows proliferation of human melanoma cells [158]. Hence careful selection requirements of TLR agonists are needed for the treatment of cancer and chronic infection either directly or by vaccination. Even though there has been tremendous progress in the development of TLR antagonists, yet efforts are needed to address the development of TLR antagonists, as some antagonist might inhibit several TLRs and therefore may provide unwanted immuno suppression. Furthermore, few TLR agonist and antagonist can induce protective or curative responses depending on the experimental model [6].

TLR therapeutic drugs have been a matter of debate, due to the differences observed in expression, function and regulation of different TLRs, for instance the key differences observed in human and mouse TLR8 [85,159], thereby animal models may not be sufficient in designing new therapeutic approaches. It is noteworthy to mention that inbred strains are being used to carry out animal studies which have less genetic diversity compared to human. In addition to this, TLR7 agonist stimulation results in widely varying induction of proinflammatory cytokines in humans. Hence there will be large margin of errors to be considered while prescribing dosage and safety windows for clinical trials.

Despite these challenges, we hope that available clinical data and the results of ongoing clinical trials support use of therapeutic targeting of intracellular TLR agonists to prevent uncontrolled viral infection and limit inflammation in several diseases.
